# Effects of gamma radiation combined with cinnamon oil on qualities of smoked salmon slices inoculated with *Shewanella putrefaciens*

**DOI:** 10.1002/fsn3.608

**Published:** 2018-03-13

**Authors:** Fei Lyu, Fei Gao, Yuting Ding

**Affiliations:** ^1^ Department of Food Science and Technology Zhejiang University of Technology Hangzhou China

**Keywords:** cinnamon oil, fatty acids, gamma radiation, lipid oxidation, *Shewanella putrefaciens*, smoked salmon

## Abstract

Smoked salmon slices inoculated with *Shewanella putrefaciens* were untreated (CK) or treated with 2 kGy gamma radiation (G), 1% (v/v) cinnamon oil (C), or the combination of them (G+C), and then packaged and stored at 4°C for 10 days. Microbiological and physiochemical analyses were then carried out. All treatments showed a better effect on inhibiting the increase in total viable counts, total volatile basic nitrogen, and thiobarbituric acid‐reactive substances than CK, especially the treatment of G+C. In addition, the combination treatment also showed a best effect on retarding the reduction in polyunsaturated fatty acids of salmon samples in all treatments. These results indicated that treatments of gamma radiation and cinnamon oil on salmon samples, especially the combination treatment, can be used to maintain the quality of smoked salmon slices.

## 1 INTRODUCTION

Gamma radiation is particularly valuable as an end product decontamination procedure (Andrews, Jahncke, & Mallikarjunan, 2003). Food radiation is one kind of “cold sterilization” methods, because of its remarkable reduction in microorganisms, and the low nutritional losses (Wood & Bruhn, 2000). It has been proposed to extend the chilled shelf life and ensure the hygienic quality of food products by reducing microbial population (Mahmoud, 2012). However, due to its high‐energy input, radiation initiates fat oxidation and breakdown of amino acids that results in off‐odors and flavors (Brewer, 2009).

Cinnamon oil, one kind of natural essential oils, possesses strong antibacterial properties (Monika, Maria, Jolanta, & Monika, 2014). It has been proved that cinnamon oil used in fish and meat products could extend their microbial shelf life (Haute, Raes, Meeren, & Sampers, 2016). The antimicrobial properties of cinnamon oil have also received great attention in the literature (Chang, Chen, & Chang, 2001; Helander et al., 1998; Kim, Park, & Park, 2004). Although essential oils (including cinnamon oil) are proved to be safe (Lambert, Skandamis, Coote, & Nychas, 2001), their use is often limited by the strong odor/taste they impart to food products.

Salmon has always been regarded as a gourmet fish species. It is the most beneficial fishes for human health because it contains the most omega‐3 unsaturated fatty acids (Simopoulos, 1991). Cold‐smoked salmon is often consumed as RTE (ready to eat) food product with no heat treatment (Montazeri, Himelbloom, Oliveira, Leigh, & Crapo, 2013). Although the processing of salting and smoking of smoked salmon can minimize the risk of food‐borne hazards and spoilage, the microbiology of smoked salmon was still considered in the previous reviews (Arvanitoyannis, 2012; Løvdal, 2015; Sikorski & Kałodziejska, 2002). Previous studies showed that *Shewanella putrefaciens* (*S. putrefaciens*) has very large spoilage potential in experiments conducted aerobically in medium with cold‐smoked salmon extracts (Gram & Huss, 1996; Hansen & Huss, 1998; Stohr, Joffraud, & Leroi, 2001).

*Shewanella putrefaciens* is a marine, Gram‐negative bacterium, which is of importance in many areas. In food products, it plays a role as a spoilage bacterium due to its ability to produce volatile sulfides, amines, and the fishy‐smelling compound trimethylamine (Bagge, Hjelm, Johansen, Huber, & Gram, 2001; Gram & Huss, 1996). The action of antimicrobials on experimentally inoculated specific organisms on meat has been reported in the literature (Løvdal, 2015). The inoculated organism model was better to understand the relationship between spoilage, microbial growth, and antimicrobial techniques. Erickson, Ma, and Doyle (2015) reported that microbial spoilage of salmon occurred and was often accompanied by unpleasant aromas during extended refrigerated storage. The combination of different antimicrobial methods has been developed to inhibit microbial growth and maintain food quality. However, little work has been conducted on the effects of gamma radiation combined with cinnamon oil. Therefore, smoked salmon slices were inoculated with *S. putrefaciens*, and the combination of gamma radiation and cinnamon oil was used as a process to inhibit microbial growth and maintain qualities of the smoked salmon slices.

## 2 MATERIALS AND METHODS

### 2.1 Samples preparation

Smoked salmon slices were bought from online shopping mall and transported into our laboratory under low‐temperature conditions within one day.

Salmon slices were immersed into 10^5^ CFU/ml *S. putrefaciens* solution at 30°C for 5 min, and then, the inoculated salmon slices were stored at 4°C for 2 h. Samples were divided into four groups randomly. The first group of samples untreated was used as control (CK), the second group was 1% (v/v) cinnamon oil (C), the third group was treated with 2 kGy gamma radiation (G), and the fourth group was treated with 2 kGy gamma radiation and 1% (v/v) cinnamon oil (G+C). Salmons of C and G+C were immersed in cinnamon oil solutions at 4°C for 10 min to make the final cinnamon oil concentration of 1% (v/v), and then, groups of G and G+C packaged with sealed ice bags were transported into the Institute of Crop and Nuclear Technology Utilization, Zhejiang Academy of Agricultural Sciences, China, as soon as possible, and then radiated. Samples were gamma‐radiated using ^137^Cs irradiator at a dose rate of 1.0 kGy/h, and the actual dose was within ± 2.0% of the target dose after dosimetry. The dose rate was established using National Physical Laboratory (Middlesex, UK) dosimeters. During radiation, all samples were maintained lower temperature using sealed ice bags covering. After radiation, all samples were transported to our laboratory, packed into the sealed general packaging bags, and stored in a refrigerator at 4°C for analysis.

### 2.2 Microbiological determination

The total viable counts (TVC) were determined on method of Gram and Huss (1996).

### 2.3 pH determination

The pH value was determined using a digital pH‐meter (PHS‐3C, Precision Scientific Instrument Co., Ltd, Shanghai, China) at ambient temperature. A sample of 10 g was homogenized in 90 ml deionized water, and the mixture was filtered.

### 2.4 TVB‐N determination

Total volatile basic nitrogen (TVB‐N) of samples was determined based on the method of Antonacopoulos and Vyncke (1989). The TVB‐N values (mg N/100 g of fish) of samples were determined according to consumption of sulfuric acid.

### 2.5 TBARS determination

Thiobarbituric acid‐reactive substances (TBARS) were determined based on the previous methods by Ke, Cervantes, and Robles‐Martinez (1984). The TBARS value was expressed as micromoles MDA per kilogram samples and was calculated from the standard curve of malonaldehyde.

### 2.6 Total lipid extraction and fatty acid composition determination

Lipid in salmon slices was extracted by the following method (Folch, Lees, & Sloane Stanley, 1957). A sample of 5 g was homogenized in the mixture of chloroform and methanol (2:1, v/v). Nonlipid impurities were removed using 4 ml of 0.88% aqueous KCl (w/v). Then, the upper layer was removed and the lower layer contains the dried lipid extract. Total lipids of samples were measured gravimetrically. The lipid extract was then used as an analysis of fatty acids.

Fatty acid methyl esters (FAME) were prepared and determined using the gas chromatograph–mass spectrometer (Trace 1300‐ISQ, Thermo, USA). TG‐5MS chromatograph column (dimensions: 30 m × 0.25 mm × 0.25 μm, Thermo Scientific, USA) was employed for chromatographic separation. The oven temperature rose from 50–150°C at a rate of 40°C/min and reached a peak temperature of 230°C at 2°C/min. A sample of 1 μl was injected into the column with the carrier gas at a flow rate of 1.5 ml/min. Data were collected and analyzed using the Chromatography Data System.

### 2.7 Statistical analysis

Experiments were performed in triplicate. All data were subjected to analysis of variance (ANOVA). Significant differences between mean values were determined using Duncan's multiple range test (*p *<* *.05). Statistical analyses were performed using SPSS (SPSS Inc., Chicago, USA).

## 3 RESULTS AND DISCUSSION

### 3.1 Microbiological analysis

**Figure 1:**
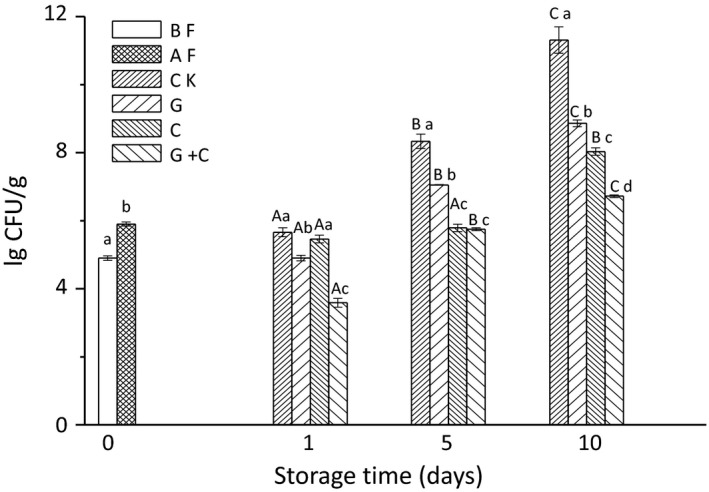
Changes in TVC in salmon slices treated with gamma radiation and cinnamon oil during storage. BF, salmon slices at the beginning without inoculating *S. putrefaciens*; AF, salmon slices at the beginning after inoculating *S. putrefaciens*; CK, untreated salmon slices group; G, salmon slices group treated with 2 kGy gamma radiation; C, salmon slices group treated with 1% (v/v) cinnamon oil; G + C, salmon slices group treated with 2 kGy gamma radiation and 1% (v/v) cinnamon oil. Values were expressed as mean ± *SD* (*n* = 3). Bars with the different letter (a–c) are significantly different within the same treatment on different storage days (*p* < .05); Bars with different letters (a–d) are significantly different within the different treatments on the same storage days (*p* < .05).

Changes in TVC in salmon slices are shown in Figure 1. TVC of salmon increased from 4.9 lg CFU/g at the beginning to 5.9 lg CFU/g after inoculated *S*. *putrefaciens*. It indicated that *S. putrefaciens* was successfully inoculated in salmon slices. During storage, an increase of TVC in all groups was observed, especially in the samples of CK. TVC of 6 lg CFU/g is often used to suggest the end of shelf life of a fish product in food industries (Gudrun et al., 2010). The values of the samples of G+C were close to and reached this level during storage; however, other groups were much higher than this level. It indicated that combination treatment of G+C had a significantly greater antimicrobial activity than C and G. The ability of radiation and cinnamon oil to prevent growth of microorganisms in food has been confirmed by other authors (Cheok et al., 2017; Haute et al., 2016). Huq, Vu, Riedl, Bouchard, and Lacroix (2015) reported that the cinnamon oil significantly improved the radiosensitivity of *L. monocytogenes*. Altan and Turan (2016) studied synergistic effect of freezing and irradiation on bonito fish and indicated that fewer bacteria were detected in irradiation as compared with control samples. In addition, when radiation is used in combination with antimicrobials, such as cinnamon oil, the antibacterial effect is strengthened through synergistic action (Huq et al., 2015). These studies indicated that the technology of radiation combined with cinnamon oil significantly reduced the growth of microorganism, which were similar to our work.

### 3.2 pH value analysis

The pH analysis during storage is a rapid and often used method for evaluating fish freshness (Truong, Buckow, Nguyen, & Stathopoulos, 2016). The pH of fish muscles can strongly reflect the postmortem changes such as autolysis process and microbiological spoilage. Values of pH in fish could be increased due to the formation of putrefactive compounds and microbiological activities during cold storage.

**Figure 2:**
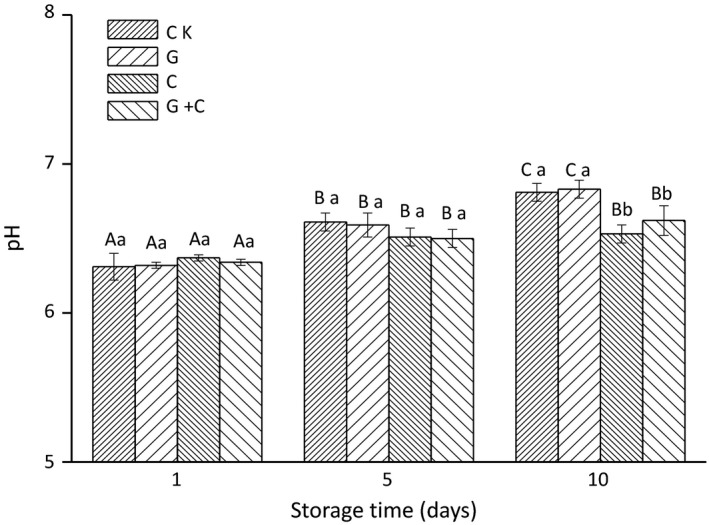
Changes in pH values in salmon slices treated with gamma radiation and cinnamon oil during storage. CK, untreated salmon slices group; G, salmon slices group treated with 2 kGy gamma radiation; C, salmon slices group treated with 1% (v/v) cinnamon oil; G + C, salmon slices group treated with 2 kGy gamma radiation and 1% (v/v) cinnamon oil. Values were expressed as mean ± *SD* (*n* = 3). Bars with the different letter (A–C) are significantly different within the same treatment on different storage days (*p* < .05); Bars with different letters (a–b) are significantly different within the different treatments on the same storage days (*p < *.05).

Changes in pH values in samples during storage are presented in Figure 2. The initial pH values of all samples on day 1 were around 6.3, and no significant difference was found between CK, G, C, and G+C. The initial pH value for salmon fillets was 6.63 and pH values ranged from 6.5 to 6.7 (Sathivel, 2005), which was similar to our results. During storage, the pH of all samples increased, especially CK and G. The increase in pH values may be due to the formation of basic decomposition products, such as alkaline volatile amines produced by spoilage bacteria. There were no significant differences among the treatments on day 1 and day 5, but on day 10, differences in G+C and C compared to others were significant (*p *<* *.05). On day 5 and day 10, no significant difference was found between C and G+C. These results indicated that cinnamon oil, especially cinnamon oil combined with radiation treatment, could slow down the increase in pH values by inhibiting the activity of bacteria.

### 3.3 TVB‐N analysis

For fish, protein and nonprotein nitrogenous compounds are greatly due to microbiological activity, which produce total volatile basic nitrogen (TVB‐N). TVB‐N is a much better indication of fish corruption, including measurement of trimethylamine, dimethylamine, ammonia, and other compounds associated with seafood spoilage (Goulas & Kontominas, 2006; Ryou, Titlow, Mays, Bae, & Kim, 2016). TVB‐N values could increase curvilinearly or linearly with time, and a level of 35–40 mg N/100 g is the limit for evaluating spoilage (Lakshmanan, 2000).

**Figure 3:**
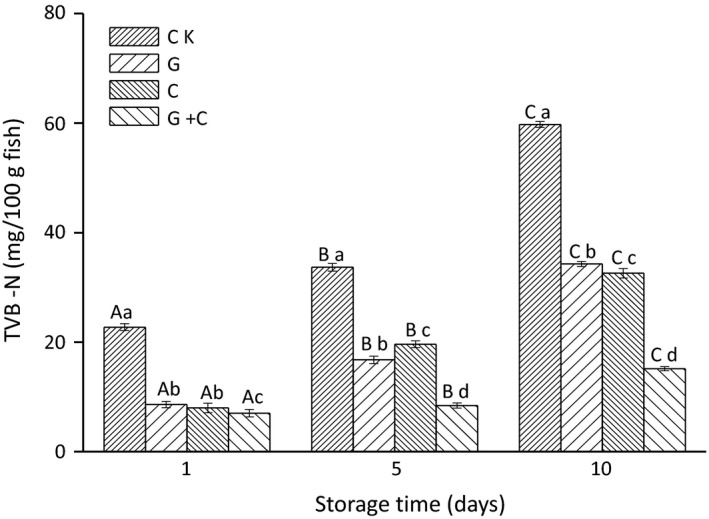
Changes in total volatile bases nitrogen (TVB‐N) in salmon slices treated with gamma radiation and cinnamon oil during storage. CK, untreated salmon slices group; G, salmon slices group treated with 2 kGy gamma radiation; C, salmon slices group treated with 1% (v/v) cinnamon oil; G + C, salmon slices group treated with 2 kGy gamma radiation and 1% (v/v) cinnamon oil. Values were expressed as mean ± *SD* (*n* = 3). Bars with the different letter (A–C) are significantly different within the same treatment on different storage days (*p* < .05); Bars with different letters (A–D) are significantly different within the different treatments on the same storage days (*p < *.05).

TVB‐N changes in all samples during storage are presented in Figure 3. The values of CK increased from 22.96 mg N/100 g on day 1 to 59.92 mg N/100 g on day 10 of storage. The TVB‐N values of G, C, and G+C groups were less than 10 mg N/100 g on day 1, which were within recommended ranges of TVB‐N in fresh fish (5–20 mg N/100 g). After 10 days of storage, the values reached 34.16, 32.48, and 15.12 mg N/100 g for samples of G, C, and G+C, respectively. TVB‐N of G+C was much less than samples of C and G. The significantly lower TVB‐N in G+C may be attributed to the strong antimicrobial activity of combination of radiation and cinnamon oil, which might inhibit both autolytic reactions and muscle protein deterioration by microorganisms.

### 3.4 TBARS analysis

The TBARS index is widely used for the assessment of lipid oxidation (Berry, 1973). It has been suggested that a maximum TBARS value, indicating the good quality of fish, is 5 mg malonaldehyde (MDA)/kg, while fish may be consumed up to the level of 8 mg MDA/kg (Sallam, 2007).

**Figure 4:**
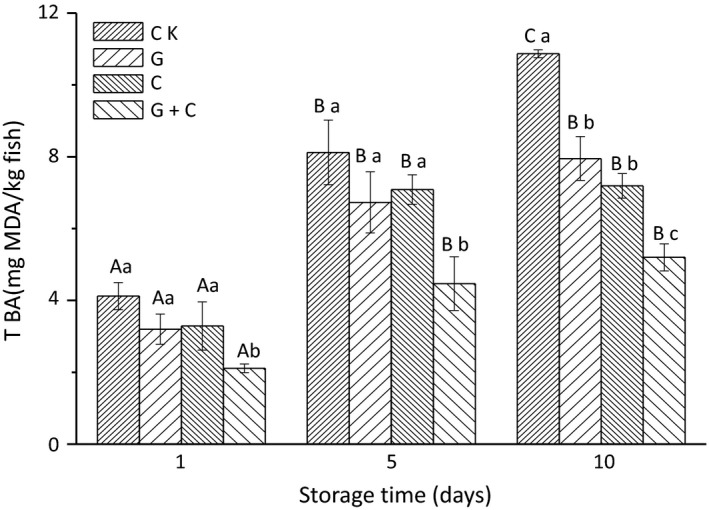
Changes in thiobarbituric acid reactive substances (TBARS) in salmon slices treated with gamma radiation and cinnamon oil during storage. CK, untreated salmon slices group; G, salmon slices group treated with 2 kGy gamma radiation; C, salmon slices group treated with 1% (v/v) cinnamon oil; G + C, salmon slices group treated with 2 kGy gamma radiation and 1% (v/v) cinnamon oil. Values were expressed as mean ± *SD* (*n* = 3). Bars with the different letter (A–C) are significantly different within the same treatment on different storage days (*p* < .05); Bars with different letters (a–c) are significantly different within the different treatments on the same storage days (*p < *.05).

TBARS changes in salmon slices during storage are presented in Figure 4. On day 1, TBARS values of CK, C, G, and G+C were 4.06, 3.68, 3.56, and 2.45 mg MDA/kg, respectively. It showed that samples treated with combination of radiation and cinnamon oil had lowest TBARS value followed by C, G, and CK during whole storage period. TBARS values of the smoked salmon slices on the first day were much higher than fresh salmon fish. It should be noted that in the traditional smoking process, the TBARS values could be increased because some compounds present in the smoke can react with the thiobarbituric acid (Beltrán & Moral, 1991). In addition, the high temperatures used in the traditional smoking processes greatly influenced the formation of secondary oxidation compounds. As shown in Figure 4, there was a general trend of increment in overall TBARS values during storage. Ten days storage later, TBARS values of CK, G, C, and G+C reached 10.79, 8.26, 7.15, and 5.09 mg MDA/kg, respectively. The increasing rate of CK was higher than that of G, C, and G+C, and TBARS of C+G was the lowest.

### 3.5 Fatty acid composition analysis

Changes in fatty acid composition of all samples during storage are shown in Table 1. The content of unsaturated fatty acids (UFAs) including monounsaturated fatty acids (MUFAs) and polyunsaturated fatty acids (PUFAs) was much higher than that of saturated fatty acids (SFAs) in salmon samples. Fish, especially salmon, is one of the best sources of docosahexaenoic acid (DHA, 22:6 n‐3) and eicosapentaenoic acid (EPA, 20:5 n‐3) for human consumers (Sargent, Tocher, & Bell, 2002). The contents of DHA and EPA were ranged from 2.12% to 2.23% and 1.22% to 1.43%, respectively, on day 1. DHA was much more than EPA in the salmon samples. This is in agreement with the report of Chaijan, Benjakul, Visessanguan, and Faustman (2006) and Kolakowska, Olley, and Dunstan (2002) that DHA is usually more abundant than EPA.

**Table 1:** Changes in the fatty acid composition in salmon slices treated with gamma radiation and cinnamon oil during storage (%)

	Day 1				Day 5				Day 10			
	CK	G	C	G+C	CK	G	C	G+C	CK	G	C	G+C
C14:0	1.44 ± 0.11a	1.42 ± 0.14a	1.57 ± 0.24a	1.52 ± 0.09a	0.74 ± 0.06a	0.66 ± 0.10ab	0.52 ± 0.10b	0.57 ± 0.15ab	0.65 ± 0.11b	1.04 ± 0.20a	0.62 ± 0.19b	0.82 ± 0.09ab
C16:0	10.27 ± 0.05a	10.32 ± 0.04a	9.76 ± 0.41b	10.26 ± 0.09a	2.86 ± 0.10d	7.11 ± 0.16a	3.84 ± 0.15c	4.40 ± 0.08b	4.12 ± 0.21b	6.07 ± 0.13a	3.57 ± 0.18b	6.43 ± 0.80a
C16:1	2.31 ± 0.03a	2.31 ± 0.03a	2.01 ± 0.08b	2.27 ± 0.06a	0.76 ± 0.09a	0.82 ± 0.09a	0.98 ± 0.20a	0.75 ± 0.20a	0.94 ± 0.26ab	1.1 ± 0.21ab	0.74 ± 0.20b	1.31 ± 0.25a
C18:0	2.76 ± 0.02a	2.76 ± 0.04a	2.76 ± 0.06b	2.74 ± 0.06b	1.48 ± 0.20a	1.15 ± 0.20b	1.00 ± 0.09b	1.22 ± 0.12ab	1.33 ± 0.31b	1.87 ± 0.15a	1.01 ± 0.19b	2.04 ± 0.24a
C18:1	26.24 ± 0.03b	26.26 ± 0.14b	24.6 ± 0.6a	26.3 ± 0.5b	7.0 ± 0.11d	10.06 ± 0.08b	8.38 ± 0.26c	11.02 ± 0.86a	8.15 ± 0.29b	11.69 ± 1.19a	7.03 ± 0.75b	10.79 ± 1.76a
C18:2	12.17 ± 0.1a	12.23 ± 0.12a	12.13 ± 0.25a	12.41 ± 0.73a	3.72 ± 0.2d	4.44 ± 0.25b	4.18 ± 0.18c	6.11 ± 0.14a	2.22 ± 0.69ab	2.72 ± 0.35a	1.64 ± 0.67b	3.17 ± 0.41a
C18:3	5.34 ± 0.42a	5.52 ± 0.12a	5.48 ± 0.09a	5.53 ± 0.22a	1.73 ± 0.16c	2.12 ± 0.18bd	1.94 ± 0.22 cd	2.79 ± 0.11a	NDc	0.74 ± 0.18b	NDc	1.06 ± 0.25a
C20:5	1.32 ± 0.06ab	1.43 ± 0.1a	1.37 ± 0.1ab	1.22 ± 0.12b	NDc	0.46 ± 0.12b	NDc	0.69 ± 0.12a	ND	ND	ND	ND
C22:6	2.23 ± 0.11ab	2.34 ± 0.07a	2.32 ± 0.07ab	2.12 ± 0.16b	NDb	NDb	NDb	1.51 ± 0.28a	ND	ND	ND	ND
SFAs	14.47 ± 0.17a	14.35 ± 0.44a	13.58 ± 0.37b	14.53 ± 0.16a	5.08 ± 0.31c	8.92 ± 0.43a	5.37 ± 0.34c	6.19 ± 0.22b	6.10 ± 0.46b	8.98 ± 0.44a	5.19 ± 0.37b	9.29 ± 0.93a
UFAs	49.61 ± 0.48a	50.08 ± 0.22a	47.93 ± 0.93b	49.85 ± 0.90a	13.21 ± 0.34a	18.01 ± 0.55b	15.48 ± 0.46c	22.86 ± 0.52d	11.31 ± 1.15a	16.25 ± 1.16b	9.41 ± 1.53a	16.33 ± 2.32b
MUFAs	28.55 ± 0.04a	28.57 ± 0.12a	26.63 ± 0.67b	28.57 ± 0.51a	7.76 ± 0.20c	10.88 ± 0.02a	9.36 ± 0.36b	11.77 ± 0.86a	9.09 ± 0.52b	12.79 ± 1.31a	7.78 ± 0.88b	10.44 ± 2.42a
PUFAs	21.06 ± 0.44a	21.51 ± 0.18a	21.3 ± 0.34a	21.28 ± 1.20a	5.44 ± 0.19c	7.13 ± 0.54b	6.12 ± 0.10c	11.09 ± 0.46a	2.22 ± 0.70b	3.46 ± 0.53a	1.64 ± 0.67b	4.23 ± 0.44a
n‐3	8.89 ± 0.50a	9.28 ± 0.28a	9.17 ± 0.24a	8.87 ± 0.47a	1.73 ± 0.16c	2.58 ± 0.30b	1.94 ± 0.22c	4.98 ± 0.38a	NDb	0.74 ± 0.18a	NDb	1.06 ± 0.25a
n‐6	12.17 ± 0.1a	12.23 ± 0.12a	12.13 ± 0.25a	12.41 ± 0.73a	3.72 ± 0.2d	4.44 ± 0.25b	4.18 ± 0.18c	6.11 ± 0.14a	2.22 ± 0.69ab	2.72 ± 0.35a	1.64 ± 0.67b	3.17 ± 0.41a

CK, untreated salmon slices group; G, salmon slices group treated with 2 kGy gamma radiation; C, salmon slices group treated with 1% (v/v) cinnamon oil; G+C, salmon slices group treated with 2 kGy gamma radiation and 1% (v/v) cinnamon oil. Values were expressed as mean ± SD (*n* = 3). The values were expressed as mean ± SD (*n* = 3). Values with different letters (a–d) are significantly different within different treatments on the same storage days (*p* < .05). SFAs, saturated fatty acids; UFAs, the unsaturated fatty acids; MUFAs, the monounsaturated fatty acids; PUFAs, polyunsaturated fatty acids. *n*‐3, *n*‐3 unsaturated fatty acids; *n*‐6, *n*‐6 unsaturated fatty acids.

After 5 and 10 days of storage, SFAs and UFAs in all samples decreased significantly. The reason might be due to the lipid oxidation during storage resulted in the reduction in SFA and UFA contents. As mentioned in Figure 4, TBARS of all samples on day 5 and day 10 were increased obviously as compared that on day 1, indicating that lipid in the salmon was oxidized during storage. Lipid oxidation increased with the storage time was also reported in the literature (Flick, Hong, & Knobl, 1992). UFA contents particularly PUFAs in salmon samples obviously decreased with the extension of storage time as compared with SFAs. This is in agreement with Pirestani, Sahari, and Barzegar (2010). Shono and Toyomizu (1971) reported that PUFAs in fish muscle decreased with oxidation, C22:6 acid decrease being most predominant, while SFAs and MUFAs showed comparatively little decrease, C16:0 acid decrease being almost negligible. Changes in fatty acids constituting lipids should be attributed not only to oxidative deterioration but also to enzymatic hydrolysis. As in fish lipids undergo hydrolysis into free fatty acids even on iced storage, it was possible that PUFAs and MUFAs undergo oxidation to a greater extent than SFAs, resulting in the decrease in PUFA and MUFA contents (Chauke, 2008). In addition, UFAs are quite susceptible to oxidative process, because the hydrogen of carbons adjacent to double bonds can be replaced by radical species with higher reactivity (Cosgrove, Church, & Pryor, 1987; Porter, Caldwell, & Mills, 1995).

As concerned for radiation and cinnamon oil treatments, the decrease of PUFAs in G+C samples was lowest in all samples. On day 5, contents of DHA in G+C samples were 1.51%, while that in CK, C and G had not been detected. It indicated that combination of radiation and cinnamon oil could prevent or prolong the degradation of fatty acids. Previous study reported that cinnamon oil had an antioxidant property and blocked lipid peroxidation of tissue lipids, especially polyunsaturated fatty acids (Keshvari, Asgary, Jafariandehkordi, Najafi, & Ghoreyshiyazdi, 2013). Furthermore, it was reported that the reduction of microorganism in foods could result in a decrease in oxidation rate of fatty acids (Bijl, Wolf, Schaap, & Visser, 2001). The present work showed that radiation combined with cinnamon oil treatment significantly reduced the growth of microorganism, which might result in the decrease in UFA oxidation.

The salmon inoculated with *S. putrefaciens* showed an increasing trend of TVC, pH, TBARS, and TVB‐N values during the storage at 4°C, but that of samples treated with radiation and cinnamon oil increased slowly compared to control samples, especially the combination of the two processes. Similarly, the decomposition rate of fatty acids had significant differences in samples between treatment and control samples. These results indicated that the combination of radiation and cinnamon oil was effective in delaying deterioration of smoked salmon.

## ETHICAL STATEMENTS

The authors declare that they do not have any conflict of interest. This study does not involve any human or animal testing. Written informed consent was obtained from all study participants.

## CONFLICT OF INTEREST

None declared.
